# The Effect of the Tongyang Huoxue Recipe (TYHX) on the *I*_to_/*I*_Kur_ in Ischemia/Reperfusion Sinoatrial Node Cells

**DOI:** 10.1155/2022/4114817

**Published:** 2022-12-20

**Authors:** Yanli Wang, Qiaomin Wu, Jinfeng Liu, Ruxiu Liu

**Affiliations:** Guang'anmen Hospital, China Academy of Chinese Medical Sciences, Beijing 100053, China

## Abstract

**Background:**

The transient outward potassium current (*I*_to_) and the ultrarapid delayed rectifier potassium current (*I*_Kur_) are major potassium currents involved in the repolarization process of sinoatrial node cells (SNCs).

**Methods and Results:**

The SNCs of neonatal rats were divided into control, ischemia/reperfusion (I/R), I/R+blank serum, and Tongyang Huoxue recipe (TYHX) serum groups. *I*_to_ and *I*_Kur_ were recorded using the whole cell patch-clamp technique, and the current-voltage (I-V), steady-state activation (SSA), steady-state inactivation (SSI), and recovery from inactivation (RFI) curves were plotted, respectively. Compared to the control group, both the peak current density and the current density at the voltage of *I*_to_ and *I*_Kur_ decreased obviously in SNCs after simulated I/R, the SSA curves moved right, and the SSI curves moved left. After TYHX was added to the extracellular solution of SNCs, both the peak current density and the current density at the voltage of *I*_to_ and *I*_Kur_ increased significantly, the SSA curves moved left, and the SSI curves moved right with a significant difference of *V*_1/2_. The recovery from the *I*_Kur_ RFI curves was slightly restored, and the *I*_to_ curves did not change.

**Conclusions:**

TYHX increases the peak current density, accelerates the activation, and decreases the inactivation of the *I*_to_ and *I*_Kur_. This may be the mechanism of TYHX in shortening the action potential duration of repolarization, which accelerates spontaneous pulsation.

## 1. Introduction

The sinoatrial node (SAN) is the primary cardiac pacemaker and the source of internal electrical stimulation driving the coordinated rhythmic contraction of the heart [1]. The SAN produces rhythmic cardiac pulsation due to its unique electrophysiological characteristics of the highest autonomic sinoatrial node cells (SNCs) and generates a spontaneous action potential (AP) that differs from peripheral atrial myocytes. SNCs are the active rhythmic cells of slow response potential with small AP, little resting potential, no platform, and only 0, 3, and 4 stages. The fourth stage of SNC, also named diastolic depolarization, a key to the SNC pacing activity, is unstable in spontaneous depolarization after repolarization.

The generation of action potentials in SNCs is achieved by the combined participation of various ionic currents across the cell membrane, including hyperpolarized activated cation current (*I*_f_), potassium currents (*I*_K_), calcium currents (*I*_Ca_), and sodium-calcium exchange current (*I*_NCX_) [1, 2]. The repolarization process of SNC ensures the spontaneous excitatory repetitive cycle of SAN. This inherent internal cycle enables SAN to serve the heart's usual pacing. Transient outward potassium current (*I*_to_) and ultrarapid delayed rectifier potassium current (*I*_Kur_) are critical currents involved in the repolarization process of SNCs [3, 4]. Both are 4-aminopyridine (4-AP) sensitivity currents characterized by rapid activation and inactivation [5]. The abnormal SNC ion channels and currents can lead to severe sinus bradycardia or arrest [6, 7].

Tongyang Huoxue recipe (TYHX) is an effective prescription for treating bradyarrhythmias, developed by Liu Zhiming, a master of Chinese medicine. It comprises five traditional herbs: *Astragalus membranaceus*, *ginseng*, *Rehmannia glutinosa*, *Angelica sinensis*, and *licorice* [8]. *Astragalus membranaceus* is a traditional Chinese herbal medicine used for treating cardiovascular disease. It contains flavonoids, saponins, and polysaccharides known to have antioxidant and anti-inflammatory properties [9]. *Ginseng* is one of the most widely available and used botanicals from East to West [10]. It has well-established pharmacological properties, including heart protection, vasodilation, anticoagulation, and antistress activity [11]. *Rehmannia glutinosa* has been commonly used for antiaging and disease-preventing in China. *Angelica sinensis* has a broad spectrum of biological activities, such as producing blood, antiaging, and anti-inflammation [12]. Traditional medicinal *licorice* is a popular food plant in Europe and China, which can strengthen the effectiveness or reduce the toxicity of other ingredients [13]. In the TCM prescription, the reasonable compatibility of those medicinal materials plays a role in regulating heart rate. However, little is known about how TYHX is involved in action potential at the repolarization phase. Therefore, this study explored the effects of TYHX on *I*_to_ and *I*_Kur_ in SNCs and the gating kinetic mechanism to elucidate the cellular electrophysiological mechanism of TYHX in enhancing the pacing function of SNCs.

## 2. Materials and Methods

### 2.1. Ethics Statement

This study has been confirmed to the Guiding Principles for the Care and Use of Laboratory Animals issued by the National Committee of Science and Technology of China and was approved by the Institutional Animal Care and Use Committee, Guang'anmen Hospital (IACUC Issue No. IACUC-GAMH-2021-006).

### 2.2. Experimental Animals

Twenty male Wistar rats (body weight 300-350 g) were provided by the Center for Experimental Animals of the Academy of Military Medical Sciences (license number SCXK (Army) 2007-004, Beijing, China), and 200 Wistar neonatal rats (male or female) were purchased from SiBeiFu Laboratory Animal Science and Technology Co., Ltd. (license number SCXK (E) 2011-0004, Beijing, China).

### 2.3. Experimental Materials and Solutions

Tongyang Huoxue recipe, which was provided by Jiuding Pharmaceuticals Co., Ltd. (batch lot 20091210, Hunan, China), was used to make the medicated serum. Dulbecco's modified Eagle medium (DMEM) was purchased from Invitrogen (United States); FBS and trypsin were purchased from GIBCO. 0.08% trypsin solution contained (g/L) 0.8 trypsin, 8 NaCl, 0.353 NaHCO_3_, 0.991 glucose, 0.298 KCl, and 2 hydroxyethyl piperazine ethanesulfonic acid (HEPES). The simulation ischemic solution contained (in mmol/L) 98.5 NaCl, 10 KCl, 0.9 NaH_2_PO_4_, 6 NaHCO_3_, 1.8 CaCl_2_, 1.2 MgSO_4_, 40 sodium lactate, and 20 HEPES (pH 6.8, adjusted with 1% hydrochloric acid). Simulated reperfusion solution contained (in mmol/L) 129.5 NaCl, 5 KCl, 0.9 NaH_2_PO_4_, 20 NaHCO_3_, 1.8 CaCl2, 1.2 MgSO_4_, 55 glucose, and 20 HEPES (pH 7.4, adjusted with 1 mmol/L NaOH). The internal pipette solution contained (in mmol/L) 130 KCl, 1 MgCl_2_, 5.0 Na_2_ATP, 5 ethylene glycol tetraacetic acid (EGTA), 10 HEPES, and 10 glucose (pH 7.2, adjusted with KOH), and the bath solution contained (in mmol/L) 140 NaCl, 1 MgCl_2_, 1 CaCl2, 4 KCl, 10 HEPES, and 5 glucose (pH 7.36, adjusted with KOH).

### 2.4. Isolation, Purification, and Culture of SNCs

SNCs were isolated from five Wistar neonatal rats (<1 day) for each procedure, identified, and cultured as previously described [14, 15]. Rats were immersed in 75% ethanol for 5-10 seconds and then fixed in a supine position on a sterile bench. The front thoracic wall was cut open with straight ophthalmic scissors to expose the heart. Tissue blocks (0.7 mm^3^) were collected from the venous sinus in the middle portion of the crista terminalis and root of the anterior vena cava under the dissecting microscope and placed in DMEM without fetal bovine serum. After vigorous pipetting, the tissue blocks were grasped with curved ophthalmic forceps and washed with phosphate-buffered saline (PBS). The tissue blocks were then immersed in PBS and cut into chylomicron. The supernatant was aspirated, and the minced tissue pieces were digested with trypsin (0.8 g/L, Sigma, USA) for 5 min in a 37°C water bath under constant shaking, pipetted vigorously for 1 min, and precipitated. The supernatants were collected and transferred to 50 mL centrifuge tubes containing 20 mL of DMEM with 15% FBS. The precipitated tissue pieces were digested again, following the same procedure described above until they were completely digested. The samples were then filtered through 400-mesh metal sieves. The filtrates were transferred to centrifuge tubes and centrifuged at 940 r/min^−1^ for 7 min. After centrifugation, the supernatants were discarded, and the cells were resuspended in a medium culture dish at a density of 1 × 10^5^ cells/L. The single cell suspension was inoculated into six small Petri dishes, divided into the control group, the I/R group, the blank serum group, and the TYHX serum group (100 *μ*L). Then, they were taken into the incubator with 95% O_2_ and 5% CO_2_ at 37°C for cell attachment, and the DMEM was changed regularly. Fibroblasts were removed through differential adherence speed, and the remaining cells were spindle-shaped, which would be SAN cells.

### 2.5. Establishment of a Simulated I/R Model in SNCs

The I/R model was constructed according to the method developed by Koyama et al. [16] and our prophase research. Ischemia was simulated by oxygen-glucose deprivation (OGD), whereas reperfusion was simulated by restoring the oxygen and sugar supply. The culture medium was replaced by the presaturated simulated ischemia solution in 95% N_2_+5% CO_2_ and then cultured in a cell incubator containing 95% N_2_ and 5% CO_2_ for one hour. After that, the simulated ischemia solution was replaced with the simulated reperfusion solution with DMED containing 10% FBS cultured for three hours. Then, the SAN cells were subjected to patch-clamp experiments. All five other groups received the above I/R procedure except for the normal group.

### 2.6. Preparation of Tongyang Huoxue Recipe-Medicated Serum

Twenty adult male Wistar rats were divided into two groups, the control serum group and the TYHX-medicated serum group, with ten rats in each group. Wistar rats received intragastric administration for seven consecutive days, two times a day, according to an equivalent dose of the rat (in 200 g of body surface area) and the adult (in 70 kg of body surface area) [17]. The daily dose volume of TYHX extract for an adult human is 14.5 g; it is 1.3 g extract/kg converted into the equivalent dose for a rat. Ten times the dosage of the equivalent TYHX recipe dose was used in the rat, so the rat received a daily gavage volume of 13 g extract/kg. The TYHX extract was suspension compounded with distilled water, and the blank serum group received an equal volume of distilled water. Blood was collected by an abdominal aortic method. Centrifuge blood at 2500 r/min for 25 minutes to obtain serum. Then inactivate the serum in the water at 56°C for 30 minutes and sterilized using 0.22 *μ*m microporous membrane filter. The sterile blank serum and the TYHX medicated serum were cryopreserved in the -80°C refrigerator for application.

### 2.7. Record the Potassium Current in SNCs

Patch-clamp experiments were performed as described in [18–20]. The whole-cell patch-clamp technique was used to record the potassium current using an Axopatch 700B amplifier (Axon Instruments, USA) with the pCLAMP 9.2 software (Axon Instruments, USA). Borosilicate glass patch pipettes (resistance = 3 − 5 M*Ω*) were pulled using a vertical pipette puller (Narishige PP-830, Japan). All the recordings were performed at room temperature (35°C) within 25 minutes to avoid the current rundown. The membrane capacitance was calculated using the manual whole-cell capacitance controls on the Axopatch amplifier.

The *I*_to_ and *I*_Kur_ were recorded in voltage-clamp mode under predefined protocols. To record the *I*_to_ current, the extracellular solution was added with dofetilide (5 nmol/L), CdCl_2_ (100 *μ*mol/L), tetrodotoxin (TTX, 100 *μ*mol/L), and 4-aminopyridine (50 *μ*mol/L) to block *I*_Kr_, *I*_CaL_, *I*_Na_, and *I*_Kur_ channels, respectively. *I*_to_ was recorded in voltage-clamp mode with 300 ms pulses from a holding potential of -80 mV to test potentials between -40 and +50 mV in steps of 10 mV. To record the *I*_Kur_ current, the extracellular solution was added with dofetilide (5 nmol/L), CdCl_2_ (100 *μ*mol/L), tetrodotoxin (TTX, 100 *μ*mol/L), and BaCl_2_ (200 *μ*mol/L) to block *I*_Kr_, *I*_CaL_, *I*_Na_, and *I*_K1_ channels, respectively. *I*_Kur_ was recorded in voltage-clamp mode with 2000 ms pulses from a holding potential of -80 mV to test potentials between -50 and +50 mV in steps of 10 mV. Each voltage pulse of *I*_to_ and *I*_Kur_ was preceded by a depolarization potential of -40 mV for 20 ms to inactivate Na^+^ currents. To learn more about gating mechanism of *I*_to_ and *I*_Kur_, steady-state activation (SSA) and steady-state inactivation (SSI) curves were generated by the Boltzmann function, *y*(*V*_*m*_) = 1/(1 + exp [(*V*_*m*_–*V*_1/2_)/k]), where *y* is the normalized current, *V*_*m*_ is the membrane potential, *V*_1/2_  is the voltage at midpoint of the available channels which are activated or inactivated, and *k* is the slope factor of curves. The curves of recovery from inactivation were obtained by fitting the time course data to a simple exponential function.

### 2.8. Statistical Analysis

The data obtained were processed using Clampfit version 10.4 (Axon Instruments) and Origin version 8.0 (Micocal Software), and the measured data are expressed as mean ± SD. Multigroup comparisons were performed using one-way analysis of variance (ANOVA), and continuous univariate data were analyzed using a Student's *t* test. Steady-state activation and inactivation curves were fit with the Boltzmann function to obtain slope factor (*k*) and midpoint (*V*_1/2_ ). The time-dependent recovery from the inactivation was fit using a first-order exponential function. *p* values less than 0.05 were considered statistically significant.

## 3. Results

### 3.1. Effects of TYHX on *I*_to_ and Its Gating Properties of SNCs

#### 3.1.1. Effects of TYHX on the Peak Current of *I*_to_

**Figure 1:**
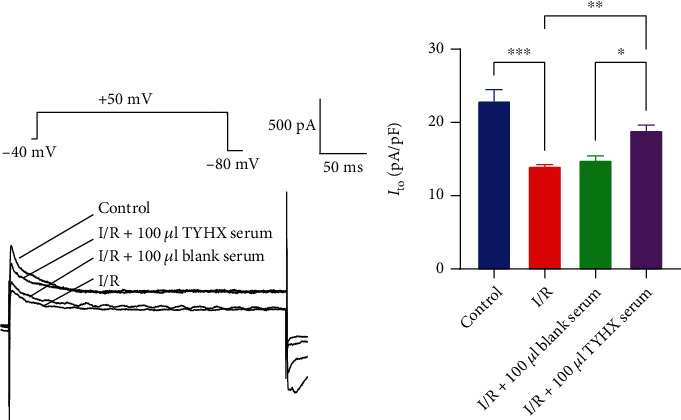
The effect of TYHX on the peak current of *I*_to_. ^∗^*p* < 0.05, ^∗∗^*p* < 0.01, and ^∗∗∗^*p* < 0.001.

The peak current of *I*_to_ of SNC was found. To explore the effects of TYHX on the potassium current of SNC, we applied blank serum and TYHX-medicated serum to SNC. A significant reduction in the *I*_to_ current can be observed in the I/R group at the peak density (Figure 1(a)). After adding 100 *μ*L blank serum, there was no obvious change at the *I*_to_ current's peak density. TYHX-medicated serum application increased the peak density of *I*_to_ current from 14.08 ± 0.71 to 18.97 ± 1.96 pA/pF, with significant change (Figure 1(b)). The results demonstrate that the simulated I/R reduced the peak current density of the *I*_to_ in the SAN cells. TYHX can increase the peak current density of the *I*_to_ and reverse the I/R-induced *I*_to_ changes in SAN cells.

#### 3.1.2. Effects of TYHX on the Current and Current-Voltage (I-V) Curves of the *I*_to_

**Figure 2:**
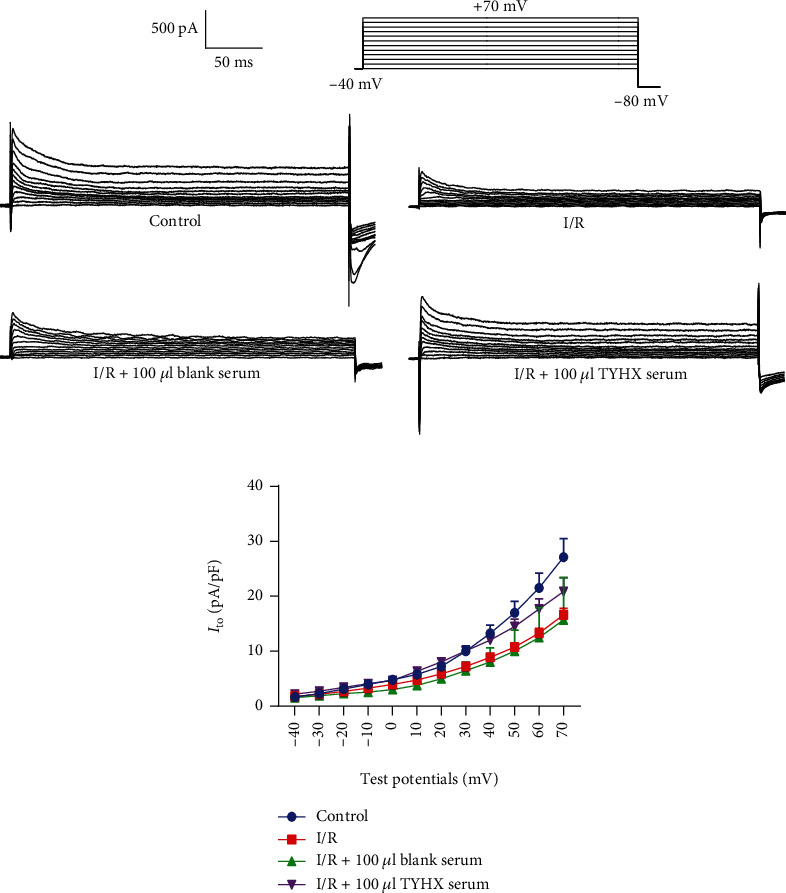
The effects of TYHX on current-voltage relationship for *I*_to_. (a) Current traces of *I*_to_ current. (b) The current-voltage curves of the *I*_to_.

Figure 2(a) shows the *I*_to_ current recorded after stimulation between voltages of -40 mV and +70 mV (10 mV depolarizing steps) from a holding potential of -80 mV. The *I*_to_ values corresponding to the above voltages were measured in each group, and *I-V* curves were constructed. The results showed (Figure 2(a)) that the current density increases as the stimulus pulse moves toward depolarization and shows a characteristic outward rectification.  Figure 2(b) shows the function of TYHX on the current-voltage relationship of the transient outward current. After simulated I/R, the current densities in the SNCs decreased under each voltage, with statistical significance above the voltages of +20 mV (*p* < 0.05) compared with the control group. The current densities of 100 *μ*L TYHX serum under each voltage increased to different degrees, especially between the voltages of +10 mV and +70 mV with statistical significance (*p* < 0.05) compared to the I/R group. There was no statistical difference between the blank serum group and the I/R group. The results indicated that the TYHX recipe-medicated serum could voltage dependently increase the current density of the *I*_to_ in SNCs.

#### 3.1.3. Effects of TYHX on the SSA and SSI Curves of the *I*_to_

**Figure 3:**
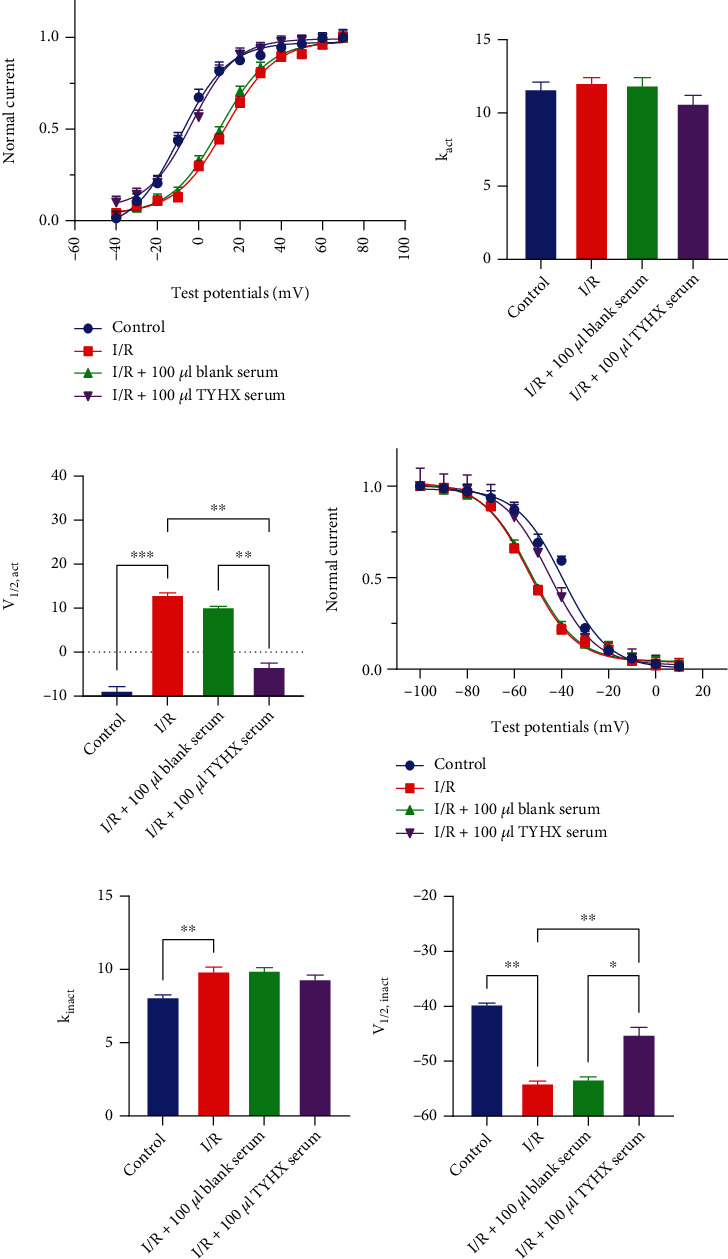
The effect of TYHX on steady-state activation and inactivation of *I*_to_. (a) The effect of TYHX on steady-state activation of *I*_to_. (b) *V*_1/2,act_ of SSA. *V*_1/2,act_ significantly rose in the I/R group, and TYHX reversed it. (c) *k* value of SSA. *k* value was no different in the three groups. (d) The effect of TYHX on steady-state inactivation of *I*_to_. (e) *V*_1/2,act_ of SSI. (f) *k* value of SSI. *k* value of SSI showed no difference in the three groups. ^∗^*p* < 0.05, ^∗∗^*p* < 0.01, and ^∗∗∗^*p* < 0.001.

We investigated the effects of TYHX on steady-state activation. Compared to the control group, the SSA curve of the *I*_to_ channel in the I/R group moved right of the depolarization direction. Besides, *k* value (the steepness of the Boltzmann curve) was changed to 12.10 ± 0.95 from the 11.66 ± 1.93 (Figure 3(b)); *V*_1/2,act_, at which 50% of channels are activated, was changed to 13.14 ± 0.97 mV from the −8.64 ± 2.25 mV (Figure 3(c)) (*p* < 0.001). No significant difference in SSA curve, *V*_1/2,act_, and *k* value was observed between the I/R group and 100 *μ*L blank serum. After adding 100 *μ*L TYHX serum, the SSA curve moved left (Figure 3(a)) and the *V*_1/2,act_ was −3.28 ± 1.66 mV with statistical significance compared to the I/R group (*p* < 0.001) (Figure 3(c)); however, the slope factor (*k* value) was 10.67 ± 1.43 with no difference (*p* > 0.05) (Figure 3(b)). The results indicated that the simulated I/R decelerated the activation of *I*_to_ in SNCs. In contrast, the TYHX serum can accelerate the activation of the *I*_to_ and restore the current density.

In steady-state inactivation profiles, the leftward shift of the SSI in the I/R group indicated that simulated I/R accelerated deactivation. In the I/R group, the steepness of the SSI curve was changed from 8.14 ± 0.54 to 9.91 ± 0.82 (Figure 3(e)) (*p* < 0.01) and the half-maximal inactivation voltage (*V*_1/2,inact_) was changed from −39.54 ± 0.61 mV to −53.93 ± 0.89 mV (Figure 3(f)) (*p* < 0.01). There was no significant difference in SSI and *V*_1/2,inact_ change between the I/R group and blank serum. TYHX serum produced a statistically significant right shift (Figure 3(d)) with *V*_1/2,inact_ of −45.06 ± 2.19 mV (*p* < 0.05) (Figure 3(f)). The slope factor *k* value was no different between the I/R, blank serum, and TYHX serum groups (Figure 3(e)). *I*_to_ channel inactivation acceleration leads to reduced current densities of the channels under the same depolarizing voltage. The results showed that TYHX serum can slow the inactivation of the *I*_to_ and restore the current density.

#### 3.1.4. Effects of TYHX on the Recovery from Inactivation (RFI) Curves of the *I*_to_ Channel

**Figure 4:**
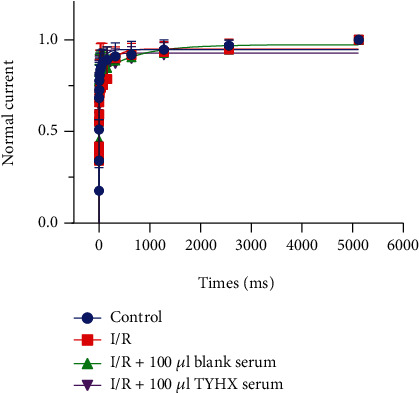
The effect of TYHX on the recovery from the inactivation of *I*_to_.

Recovery from inactivation was assessed by a standard double-pulse protocol using a recovery potential of -100 mV. Data of recovery time was then fitted with a simple exponential function. While *I*_to_ was increased in TYHX application, we observed no difference in recovery from inactivation between all groups (Figure 4).

### 3.2. Effects of TYHX on *I*_Kur_

#### 3.2.1. Effects of TYHX on the Peak Current of *I*_Kur_

**Figure 5:**
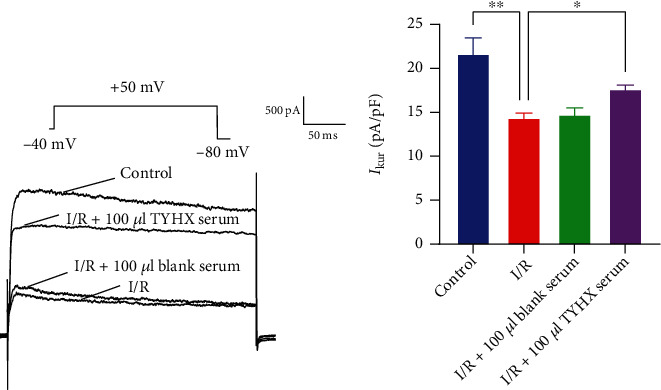
The effect of TYHX on the peak current of *I*_Kur_. ^∗^*p* < 0.05 and ^∗∗^*p* < 0.01.

The current of *I*_Kur_ was found in SNC. We determined the peak current density by measuring the peak *I*_Kur_ current as shown (Figure 5). The peak current of the *I*_Kur_ was significantly lower in the I/R group and 100 *μ*L blank serum group, and the peak current increased after adding 100 *μ*L TYHX serum (Figure 5(a)). No significant differences were found between the I/R group and 100 *μ*L blank serum group. TYHX serum application increased the peak density of *I*_Kur_ current from 14.4 ± 0.9 to 17.69 ± 1.92 pA/pF, with significant change (*p* < 0.05) (Figure 5(b)). The above results demonstrate that the simulated I/R reduced the peak current density of the *I*_Kur_ in the SAN cells. The TYHX recipe can increase the peak current density of the *I*_Kur_ in damaged SAN cells and reverse I/R-induced *I*_Kur_ changes in SAN cells.

#### 3.2.2. Effects of TYHX on the *I-V* Curves of the *I*_Kur_

**Figure 6:**
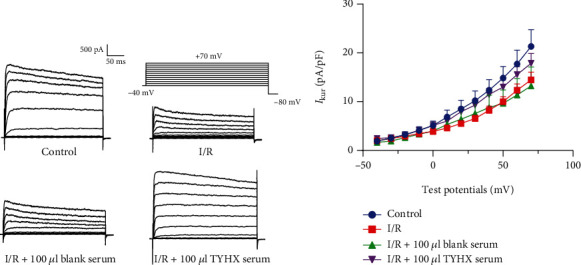
*I-V* curves of the *I*_Kur_. (a) Effect on the *I*_Kur_ current under the train of stimulation. (b) Effect on the *I-V* curves of the *I*_Kur_.

Figure 6 shows the *I*_Kur_ current recorded after stimulation between voltages of -40 mV and +70 mV (10 mV depolarizing steps) from a holding potential of -80 mV. The results showed (Figure 6) that the current density increases as the stimulus pulse moves toward depolarization and shows a characteristic outward rectification. After simulated I/R, the current densities in the SNCs decreased under each voltage, with a statistically significant difference between the voltages of +10 mV and +70 mV (*p* < 0.05) compared with the control group. After adding 100 *μ*L TYHX serum, we found that all current densities under each voltage increased to different degrees, especially between voltages of +20 mV and +70 mV, with statistical significance (*p* < 0.05) compared to the I/R group. Nevertheless, there was no statistical difference between the blank serum group and the I/R group. The results indicated that I/R leads to decreased current density. The TYHX serum could increase the current density of the *I*_Kur_ in the SNCs with a voltage-dependent manner.

#### 3.2.3. Effects of TYHX on the SSA and SSI Curves of the *I*_Kur_

**Figure 7:**
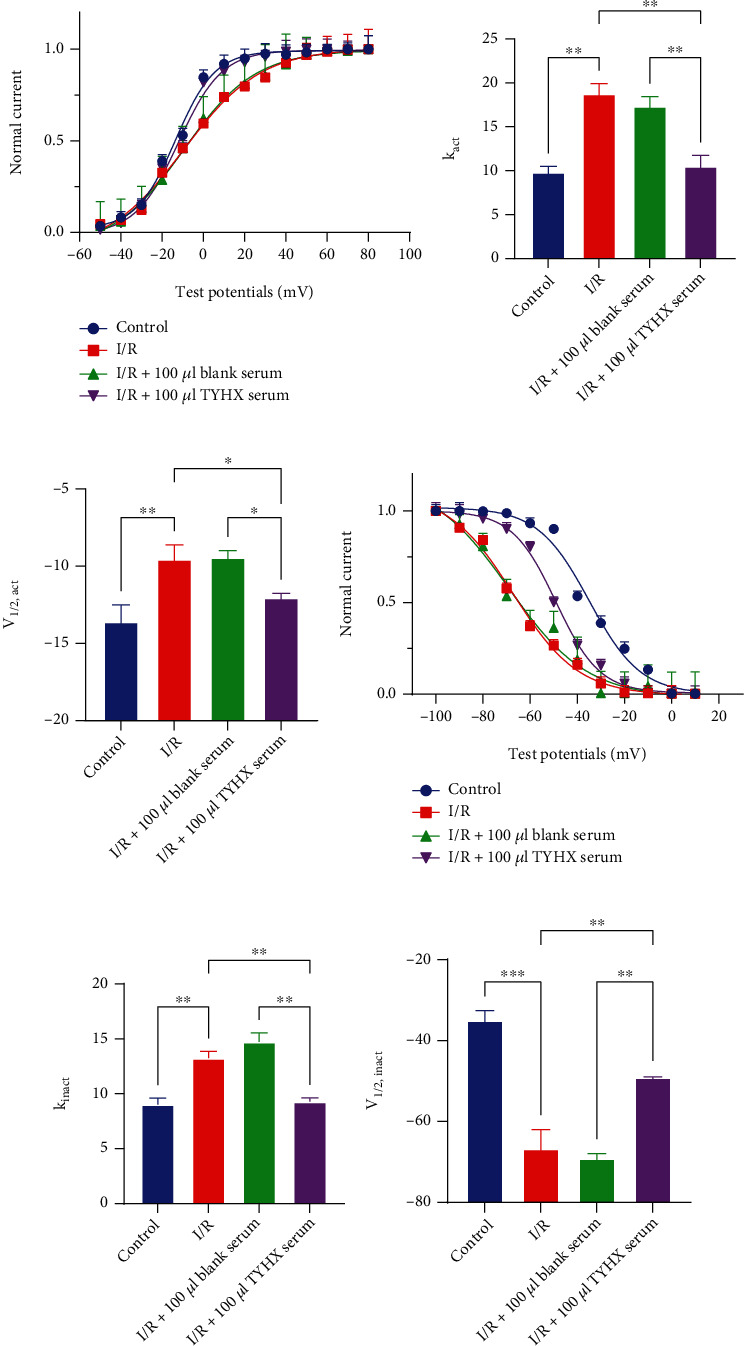
The effect of TYHX on steady-state activation and inactivation of *I*_Kur_. (a) The effect of TYHX on steady-state activation of *I*_Kur_. (b) *V*_1/2,act_ of SSA. (c) *k* value of SSA. (d) The effect of TYHX on steady-state inactivation of *I*_Kur_. (e) *V*_1/2,act_ of SSI. (f) *k*  value of SSI. ^∗^*p* < 0.05, ^∗∗^*p* < 0.01, and ^∗∗∗^*p* < 0.001.

Fitted curves of steady-state activation and inactivation were generated by the Boltzmann function. In the I/R group, the SSA curve of the *I*_Kur_ channel moved right (Figure 7(a)). In details, the slope factor *k* value changed from 9.84 ± 1.11 to 18.76 ± 2.01 (*p* < 0.01) (Figure 7(b)), and the half-maximum activation voltage (*V*_1/2,act_) was changed from −13.6 ± 1.9 mV to −9.53 ± 1.57 mV (*p* < 0.05) (Figure 7(c)) with the statistical difference compared with the control group. After adding 100 *μ*L TYHX serum, the SSA curve moved left, the *k* value changed to 10.5 ± 2.13, and the *V*_1/2,act_ changed to −12.03 ± 0.86 mV, both with statistical significance compared to the I/R group (*p* < 0.01) (Figures 7(b) and 7(c)). The results indicated that the simulated I/R decelerated the activation of the *I*_Kur_ to decrease the current density. And the TYHX serum can accelerate *I*_Kur_ activation and restore current density, thus may shorten the duration of repolarization of the SNC action potential and finally improving the autorhythmicity of SNCs.

In steady-state inactivation curves, the SSI curve moved left in the I/R group, accelerating the deactivation of *I*_Kur_ (Figure 7(d)). Comparisons between the I/R group and blank serum group showed no difference in slope factor *k* value and *V*_1/2,inact_. After adding TYHX, the SSI curve moved right, which means deactivation of *I*_Kur_ slowed (Figure 7(d)). The slope factor *k* value changed from 13.24 ± 1.08 to 9.29 ± 0.57 after adding 100 *μ*L TYHX serum (*p* < 0.01) (Figure 7(e)). The data fitted a Boltzmann equation with *V*_1/2,inact_ values of −66.67 ± 8.11 mV and −49.06 ± 0.81 mV for the I/R group and after TYHX application, respectively (Figure 7(f)). The results indicated that the simulated I/R accelerated the inactivation of the *I*_Kur_, which resulted left shift of SSI curves. On the contrary, the TYHX recipe can slow deactivation of the *I*_Kur_ and restore the current density to accelerate the repolarization.

#### 3.2.4. The Recovery from Inactivation (RFI) Curves of the *I*_Kur_

**Figure 8:**
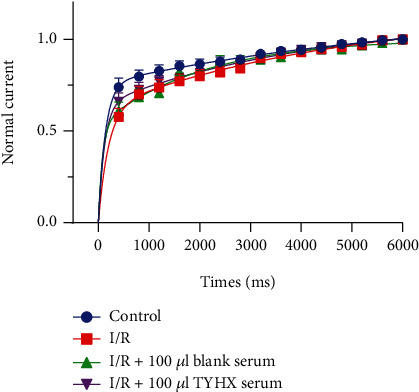
The effect of TYHX on the recovery from the inactivation of *I*_Kur_.

Recovery from inactivation is closely related to inactivation and critically determines *I*_Kur_ channel function. We observed slower recovery from inactivation in the I/R group compared to the control group, especially in the time of the former 3000 ms. The application of TYHX showed much faster deinactivation than the I/R group and blank group (Figure 8).

## 4. Discussion

Sick sinus syndrome (SSS) is an organic disease of SAN and its surrounding tissues, which leads to a series of clinical manifestations caused by pacing and impulsive efferent disturbance. It is a significant clinical refractory cardiovascular disease, manifested as bradycardia and conduction block, that can lead to the inadequate blood supply to the heart, brain, kidney, and other vital organs. The prevalence of SSS has been reported to be 1/1000 in adults over 45 years [21] and 1/600 in adults over 65 years of age [22]. Sudden cardiac arrest poses a severe threat to patients' lives, and the number of new patients with SSS is expected to increase to 172,000 annually by 2060 in the United States [21]. SSS represents more than 50% of permanent pacemaker implantations globally [23].

Natural products have been globally recognized for having broad clinical application prospects in light of their advantages with respect to multitargets, efficacy advantages, and safety. With the rapid advances of modern technology, such as the patch clamp, we can evaluate a variety of Chinese antiarrhythmic medicines or their effective ingredients. Natural compounds are widely used in traditional Chinese medicine and have been verified to act on ion channels to alter ion channel gating against arrhythmia [24]. Previously, we elucidated that natural products of astragaloside could shorten the AP duration in damaged SAN cells of neonatal rabbits, thereby increasing the expression of HCN4 and the *I*_f_ current density in a voltage-dependent manner [25]. Components of TYHX were identified by previous high-performance liquid chromatography (HPLC) studies, and five significant components were confirmed, including ferulic acid, ginsenoside Rb1, astragaloside IV, diosgenin, and catalpol [8]. These components of TYHX have great potential for therapy and prevention in multiple cardiovascular diseases, including antioxidant, antiapoptotic, and anti-inflammatory effects [26, 27]. In addition, it can play a protective role for various cells (cardiomyocytes, sinoatrial node cells, and endothelial cells) under stress conditions [28–30].

The serum pharmacology method has been frequently used to study the effects and mechanisms of Chinese drugs in vitro, as it eliminates the interferences of the physical and chemical characteristics of crude herbs in the experimental process and provides similar experimental conditions to the in vivo environment. The results showed that blank serum from rats does not significantly affect the *I*_to_ and *I*_Kur_ current density or the process of activation and inactivation compared to the I/R group. Therefore, we used the method of serum pharmacology in vitro experiments and observed the effect of mediated serum on the regulation of *I*_to_ and *I*_Kur_.

The abnormality of SNCs' ion channels and currents can lead to SSS [31], in which *I*_to_ and *I*_Kur_ are critical currents involved in action potential repolarization of SNCs [32]. Changes in *I*_to_ and *I*_Kur_ affect the action potential duration and then affect the frequency of pulsation, which is of great significance to the regulation of action potential in SNCs. *I*_to_ in mammalian ventricular and atrial cells has been shown to be generated by K^+^ outward ions that largely contribute to the formation of the AP during the early repolarization phase (phase 1) [33]. A host of molecules modulate *I*_to_, including Kv1.4, Kv4.2, and Kv4.3 channels. Some researchers have suggested that phase 1 of the AP in SAN cannot be identified. However, other studies showed that *I*_to_ significantly promoted the repolarization of rabbit sinoatrial node cells [34], and action potential duration (APD) was prolonged and current intensity was decreased in pacemaker cells when *I*_to_-sensitive blocker 4-aminopyridine (4-AP) blocked *I*_to_ [32]. *I*_Kur_ also leads to rapid repolarization of the action potential and balances the inward depolarization current of Na + and L-type Ca^2+^ channels [35]. Block of *I*_Kur_ results in a 39% prolongation of the AP and a 27% increase in cycle length [36]. Kv1.5 (KCNA5) is believed to encode the pore-forming subunit of *I*_Kur_ [37], located at or close to desmosomes in SA node tissue [36]. An age-related decrease in Kv1.5 was found in the sinoatrial node of rats [38], which may partly explain the phenomenon that the duration of AP increases with aging [39]. As SSS is an aging disease, specific evidence is increasing for age-related remodeling of the ion channels in SAN [40].

*I*_to_ and *I*_Kur_ play an important role in regulating spontaneous cardiac rhythms and heart rate. The results of the present study show that TYHX increased the *I*_to_ and *I*_Kur_ current density in the damaged SAN cells in a voltage-dependent manner. The SSA curve of the *I*_to_ and *I*_Kur_ channels was shifted to the left, indicating that the TYHX treatment accelerated channel activation. The SSI curve of the *I*_to_ and *I*_Kur_ channel was shifted to the right, which illustrates TYHX slows the inactivation of *I*_to_ and *I*_Kur_ channel. The result estimated the possibility of TYHX in shorting the depolarization phase of the action potential and improving the autorhythmicity of SAN cells.

## 5. Conclusions

Our research elucidates the mechanism of TYHX in treating SSS from the aspect of cell electrophysiology. Combining with previous studies, our group systematically and intensely studied the clinical efficacy and mechanism of TYHX in the treatment of SSS from different levels, such as theoretical basis, animal experiment, holistic effect, cell molecule, and gene expression. It proved that TYHX has multiple goals and ways, playing an essential role in providing a scientific basis for the research methods and ideas for the treatment of diseases with TCM, which can deepen the scientific connotation of famous TCM experiences.

## Data Availability

The data that support the findings of this study are available from the corresponding author upon reasonable request.
